# Basing intubation of acutely hypoxemic patients on physiologic principles

**DOI:** 10.1186/s13613-024-01327-w

**Published:** 2024-06-12

**Authors:** Franco Laghi, Hameeda Shaikh, Nicola Caccani

**Affiliations:** 1 Division of Pulmonary and Critical Care Medicine, Hines Veterans Affairs Hospital (111N) and Loyola University of Chicago Stritch School of Medicine, 60141 Hines, IL USA; 2 Department of Physiology and Pharmacology, Center for Molecular Medicine, Department of Clinical Neuroscience, Karolinska Institutet, Stockholm, Sweden

**Keywords:** Hypoxemic respiratory failure, thresholds for invasive ventilation, Intratracheal intubation, Hypoxemia, Mechanical ventilation, Critical care, Clinical decision rules

## Abstract

The decision to intubate a patient with acute hypoxemic respiratory failure who is not in apparent respiratory distress is one of the most difficult clinical decisions faced by intensivists. A conservative approach exposes patients to the dangers of hypoxemia, while a liberal approach exposes them to the dangers of inserting an endotracheal tube and invasive mechanical ventilation. To assist intensivists in this decision, investigators have used various thresholds of peripheral or arterial oxygen saturation, partial pressure of oxygen, partial pressure of oxygen-to-fraction of inspired oxygen ratio, and arterial oxygen content. In this review we will discuss how each of these oxygenation indices provides inaccurate information about the volume of oxygen transported in the arterial blood (convective oxygen delivery) or the pressure gradient driving oxygen from the capillaries to the cells (diffusive oxygen delivery). The decision to intubate hypoxemic patients is further complicated by our nescience of the critical point below which global and cerebral oxygen supply become delivery-dependent in the individual patient. Accordingly, intubation requires a nuanced understanding of oxygenation indexes. In this review, we will also discuss our approach to intubation based on clinical observations and physiologic principles. Specifically, we consider intubation when hypoxemic patients, who are neither in apparent respiratory distress nor in shock, become cognitively impaired suggesting emergent cerebral hypoxia. When deciding to intubate, we also consider additional factors including estimates of cardiac function, peripheral perfusion, arterial oxygen content and its determinants. It is not possible, however, to pick an oxygenation breakpoint below which the benefits of mechanical ventilation decidedly outweigh its hazards. It is futile to imagine that decision making about instituting mechanical ventilation in an individual patient can be condensed into an algorithm with absolute numbers at each nodal point. In sum, an algorithm cannot replace the presence of a physician well skilled in the art of clinical evaluation who has a deep understanding of pathophysiologic principles.

## Case report-vignette

**Fig. 1:**
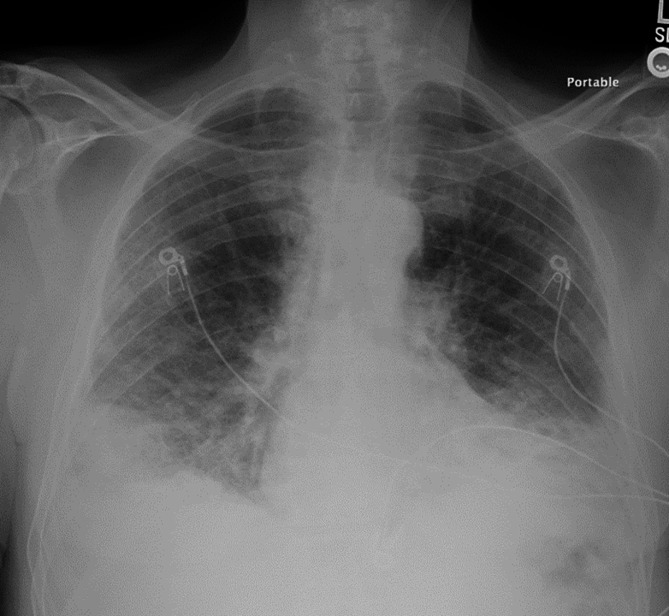
Portable chest radiograph of index case obtained on hospital admission. Bilateral mid and lower lung interstitial and airspace opacities. Right hemidiaphragm elevation with lateral lobulated contour stable when compared to previous chest imaging (not shown)

**Fig. 2:**
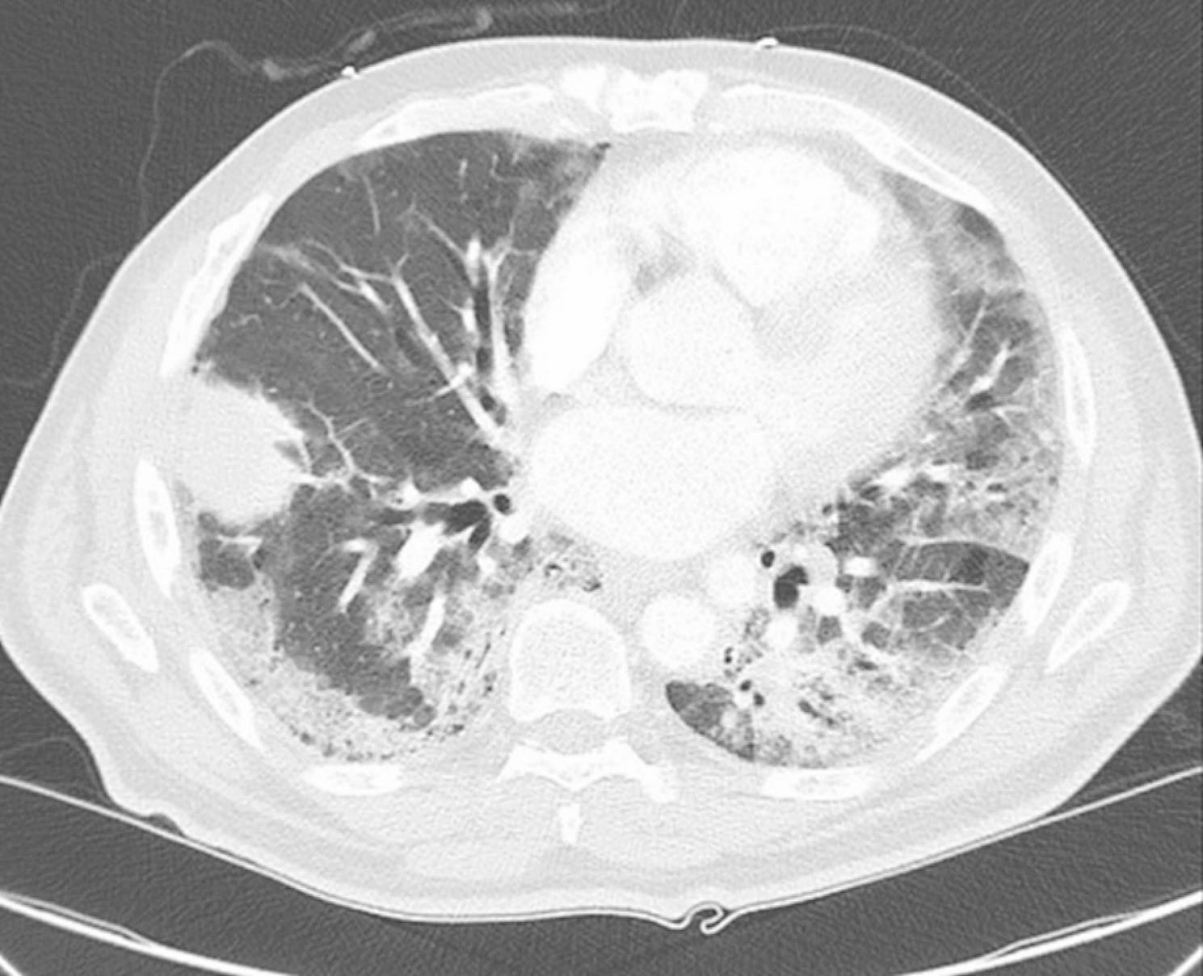
Computed tomography of the chest of index case obtained on day-2 of hospital admission: Multiple bilateral ground glass opacities and consolidations. The exam was negative for pulmonary embolus (not shown)

*A 73-years old man with history of hypertension is admitted to the hospital with cough, chills, and dyspnea on exertion. On arrival his peripheral oxygen saturation (SpO*_2_*) on room air ranges from 80 to 83%. Following administration of 4 L·min*^− 1^*oxygen by nasal cannula, SpO*_2_*increases to 94%. He is afebrile (37.5 °C), normotensive. Heart rate is 93 bpm and respiratory rate is 20 bpm. The patient is in no apparent respiratory distress. He tests positive for SARS-CoV2. Chest radiograph demonstrates bilateral mid and lower lung opacities (Fig.* 1*). Two days later he is transitioned to high-flow oxygen through nasal cannula. At times, SpO*_2_*is in the low 80s% and occasionally in the 70s%. Computed tomography of the chest demonstrates multiple bilateral ground glass opacities and consolidations(Fig.* 2*).Although tachypneic, he continues to report no respiratory distress. His mentation is normal. Should he be intubated?*

## Background

Notwithstanding that intensivists strive to support the function of all vital organs, at a fundamental level their primary goal is to ensure that a patient’s oxygenation is sufficient to avoid cerebral hypoxia. To this end, investigators initiate invasive ventilation in patients who remain hypoxemic despite implementation of noninvasive oxygenation strategies based on different oxygenation thresholds [1]. Unfortunately, these thresholds have serious limitations that can result in either overly liberal intubation (unnecessarily exposing patients to the risks of inserting an endotracheal tube [2] and of invasive ventilation [3, 4] ) or overly conservative intubation (exposing patients to the dangers of hypoxia [5–7]). The situation becomes even more perplexing when the decision to intubate is based not on presumed tissue hypoxia (as indirectly suggested by oxygenation indexes), but rather on the unclear association between poor oxygenation indexes and worse clinical outcomes [1, 8].

In this review we will first discuss intubation criteria based on oxygenation thresholds and their limitations. Then, we will discuss our approach to intubation based on clinical observations and physiologic principles. Our focus is on acutely hypoxemic patients, with or without COVID-19, who are variably tachypneic but who are neither in apparent respiratory distress nor in shock.

## Intubation criteria based on SpO_2_ thresholds

**Fig. 3:**
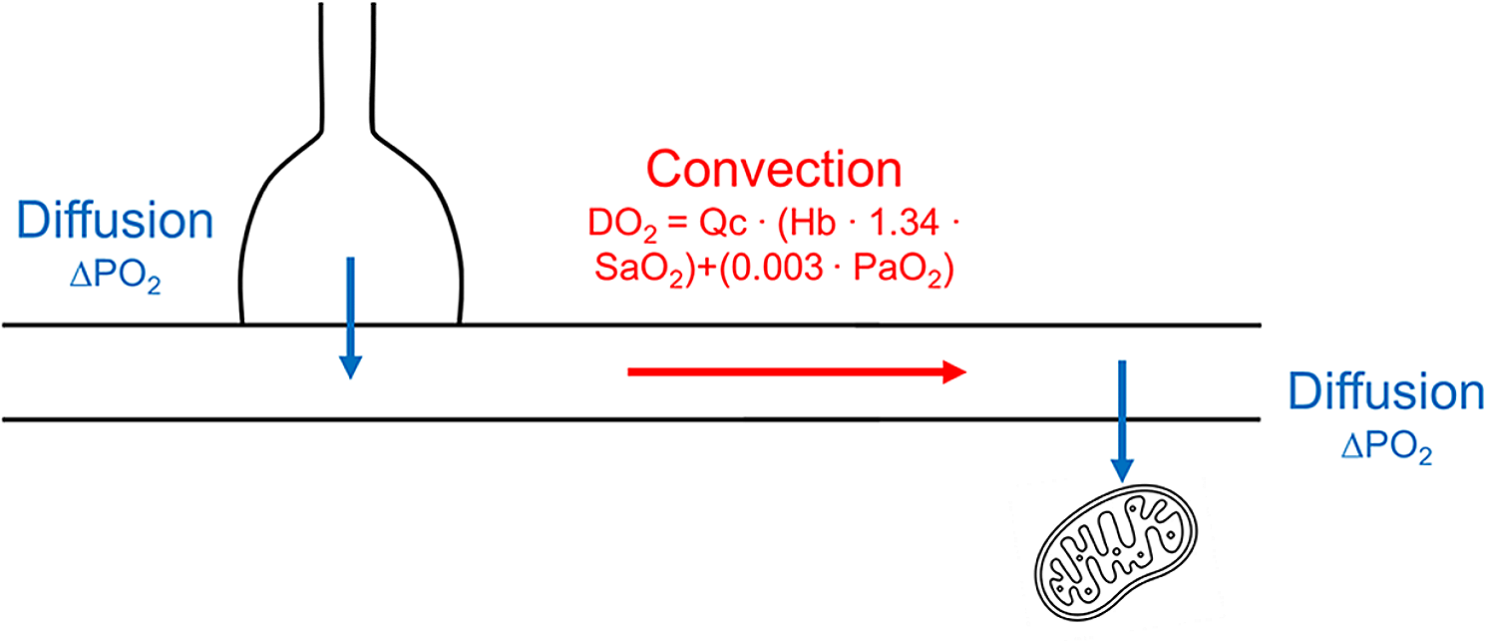
Schematic representation of the movement of oxygen from inhaled gas to tissue mitochondria. This movement requires both diffusion and convection of oxygen. Diffusion of oxygen, or diffusive oxygen delivery, is a passive phenomenon whereby the gradient in oxygen pressure (∆PO_2_) drives oxygen from the alveolus to the plasma (left blue arrow) and from the plasma to the interstitial fluid and tissue mitochondria (right blue arrow). Convective (perfusive) oxygen delivery (DO_2_) is an energy-requiring process that relies on the work performed by the respiratory and cardiac pumps to move the oxygen carried in the blood from the lungs to the peripheral tissues. Convective oxygen delivery is a function of cardiac output (Qc) and arterial oxygen content. Arterial oxygen content is mainly determined by hemoglobin (Hb) concentration and percentage saturation of hemoglobin with oxygen (SaO_2_), with only a small contribution determined by the partial pressure of oxygen (PaO_2_) (see text for details)

Some investigators recommend intubation when SpO_2_ readings are less than 92% [9–11], 90% [12–15], 88% [16], or 85% [17, 18]. Technical limitations of the devices used to record SpO_2_ confound its interpretation. Moreover, the inherent inaccuracy of SpO_2_ in estimating both the volume of oxygen transported in the arterial blood *(convective oxygen delivery)*, and the pressure gradient driving oxygen from the capillaries to the cells *(diffusive oxygen delivery)*(Fig. 3) [19, 20] cast serious doubts on the clinical utility of these thresholds.

### Drawbacks of SpO_2_ monitoring devices

**Fig. 4:**
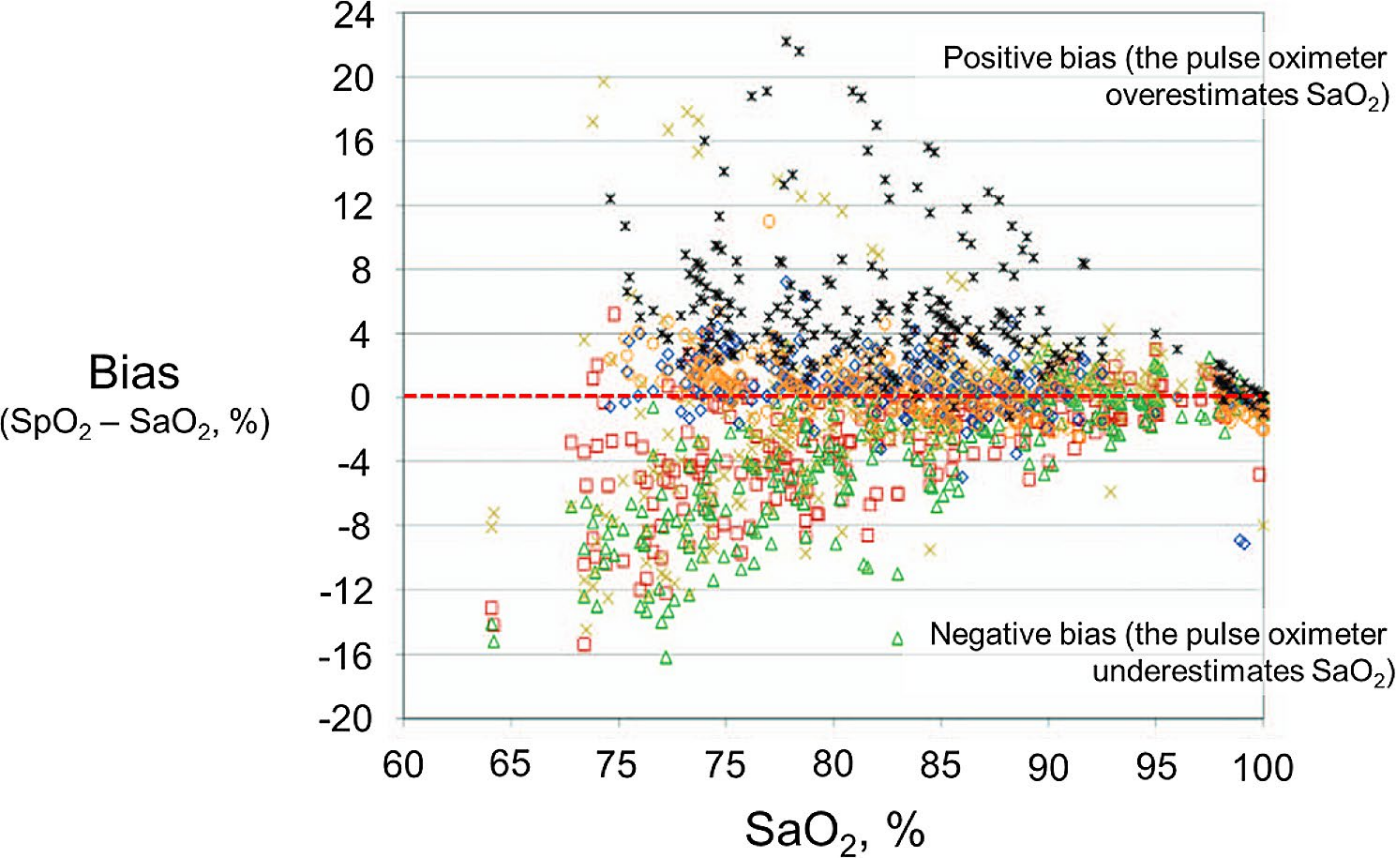
Relationship between arterial blood oxygen saturation (SaO_2_) measured with a CO-oximeter and the difference (bias) between peripheral oxygen saturation (SpO_2_) measured with six different fingertip pulse oximeters and SaO_2_. Each oximeter is indicated by a different symbol. Measurements were obtained in 22 heathy volunteers of different ethnicities during controlled laboratory hypoxia conditions. In the absence of bias, all the datapoints would rest on the red broken horizontal line (zero bias). Instead, all pulse oximeters demonstrated either positive bias (signifying overestimation of SaO_2_) or negative bias (signifying underestimation of SaO_2_). Bias worsened as subjects became more hypoxemic. These results are similar to those recorded with larger benchtop pulse oximeters [91]. (Modified from [26])

**Fig. 5:**
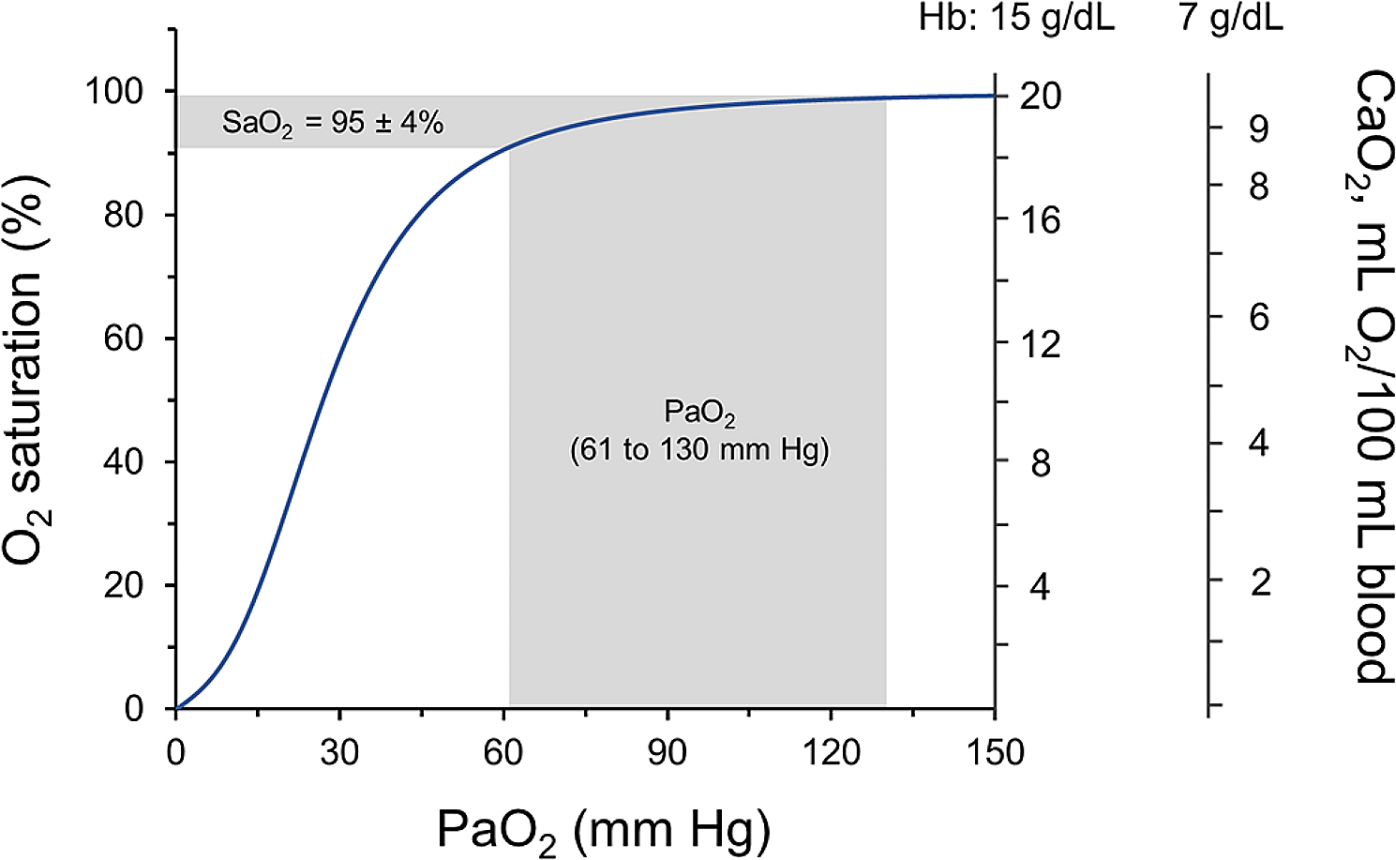
Relationship between arterial oxygen pressure (PaO_2_) and percentage saturation of hemoglobin with oxygen (SaO_2_) applicable when the pH of the blood is 7.40 and temperature is 37° C. Since oximeters have 95% confidence limits for SaO_2_ of about ± 4%, an oximeter reading of 95% could represent a PaO_2_ of 61 mm Hg (saturation 91%) or a PaO_2_ of 130 mm Hg (saturation 99%). The right vertical axes represent values of arterial oxygen content (CaO_2_) based on the common hemoglobin concertation in a healthy adult of 15 g/dL or based on the hemoglobin concentration of 7 g/dL, a hemoglobin concentration below which providers usually transfuse packed red blood cells. (See text for details)

Pulse oximetry estimates arterial oxygen saturation (SaO_2_) by illuminating the skin and measuring changes in light absorption of oxyhemoglobin (HbO_2_) and reduced hemoglobin (Hb) [21]. SpO_2_ can differ from true SaO_2_ (measured with a CO-oximeter) as much as ± 4% or more [22–26] (Fig. 4). The inaccuracy of SpO_2_ in identifying SaO_2_ combined with the sigmoid shape of the oxygen oxyhemoglobin dissociation curve (Fig. 5), has major implications for the recognition of early deterioration of gas exchange in patients with normal baseline arterial oxygen pressure (PaO_2_) [27]. This is because on the upper near-horizontal portion of the dissociation curve, large changes in PaO_2_ cause little changes in SaO_2_. For instance, with a 95% confidence limit of about ± 4%, an SpO_2_ reading of 95% could represent any PaO_2_ that starts from 130 mmHg (i.e., SaO_2_ of 99%) and deteriorates to 61 mm Hg (i.e., SaO_2_, of 91%) (Fig. 5) [28].

Pulse oximeters are less accurate in patients with increased melanin. In one of the original studies on this phenomenon, Jubran and Tobin [29] reported that in critically ill, mechanically ventilated patients, pulse oximetry is 2.45 times less accurate in Black patients. Over the last three decades, the findings of Jubran and Tobin [29] have been corroborated by multiple investigators [25, 30–32]. For instance, Burnett al [31] compared SpO_2_ vs. SaO_2_ in more than forty-five thousand patients undergoing general anesthesia. In that study, the occurrence of occult hypoxemia, defined as SaO_2_ < 88%, when SpO_2_ reading remained > 92%, was 2.1% in Blacks, 1.8% in Hispanics, and 1.1% in Whites. More recently, in a study of nearly three thousand patients with COVID-19, Crooks et al. [30] reported occult hypoxemia in 6.9% patients of mixed ethnicities, in 5.4% Black, 5.1% Asian and 3.2% White patients [30]. The large 95% confidence limits (see Table 1 in Crooks et al. [30]) indicate that pulse oximetry provides both falsely high and falsely low saturations in all ethnicities. An additional confounder is that the difference between SpO_2_ and SaO_2_ is not reproducible (in magnitude or direction) [33]. These observations raise several considerations. The inaccuracy of pulse oximeters with skin pigmentation rests on the fact that reference calibration curves continue to rely on White volunteers [34]. Pulse oximetry either overestimates or underestimates SaO_2_ in all ethnicities. It is possible that the reduced accuracy of pulse oximeters in patients with increased melanin have contributed to the increased morbidity and mortality of these patients before and during the COVID-19 pandemic [32, 35, 36].

### Drawbacks of using SpO_2_ to estimate oxygen delivery

Convective (perfusive) oxygen delivery (DO_2_) is an energy-requiring process that relies on the work performed by the respiratory and cardiac “pumps” [19]. Convective DO_2_ is the product of cardiac output (Qc) and arterial blood oxygen content (CaO_2_). The latter, in turn, is the product of SaO_2_ and Hb. (Under most circumstances the amount of oxygen dissolved in the blood is negligible.) SpO_2_ gives only an estimate of SaO_2_, that is, in turn, but one contributor to convective DO_2_. Accordingly, a given SpO_2_ estimation of SaO_2_, even if high, can be inadequate in securing sufficient convective DO_2_ to the brain if Hb or Qc are critically reduced. At the same time, a low estimation of SaO_2_ can still secure sufficient convective DO_2_ to the brain if the cardiovascular compensatory mechanisms are adequate, and Hb and SaO_2_ are not critically reduced.

The diffusion of oxygen from the alveoli to the pulmonary capillaries and from the systemic capillaries to the cells, or diffusive DO_2_, is a passive phenomenon that depends on the gradient in partial pressure of oxygen (PO_2_), tissue capillary density, and the ability of the cell to take up and use oxygen [19] (Fig. 3). The technical limitations in obtaining valid SpO_2_ recordings and the many factors that modulate the oxygen-dissociation curve (see below) make it unrealistic to use SpO_2_ to estimate diffusive DO_2_.

## Intubation criteria based on SaO_2_ thresholds

Cognizant of the limitations of SpO_2_ readings, some investigators recommend intubation of hypoxemic patients when SaO_2_ is less than 92% [37], 90% [37], 85% [38, 39] or 80% [39].

### Drawbacks of SaO_2_monitoring devices

SaO_2_ can be directly measured using core laboratory CO-oximeters, or it can be calculated using point-of-care devices [40]. CO-oximeters determine SaO_2_ spectrophotometrically. They are considered the reference standard technique to measure SaO_2_ [40]. These devices exhibit good intra-device reproducibility [41] yet, as expected, they exhibit inter-device discrepancy, both between two identical devices produced by the same manufacturer [42] or between two devices produced by different manufacturers [43]. Inter-device discrepancy increases as hypoxemia worsens [42, 43].

Point-of-care devices calculate SaO_2_ using algorithms that rely on measured parameters such as arterial pH, PO_2_ and PCO_2_ [40]. Point-of-care calculation of SaO_2_ increasingly deviates from SaO_2_ measured by CO-oximetry under hypoxemic conditions. For instance, in a study of more than three thousand samples, Gunsolus et al. [40] recorded an increase in the percent difference between measured and calculated SaO_2_ of about ± 2% when PaO2 was greater than 90 mm Hg to ± 20% or more when PaO_2_ was 50 to 60 mmHg or less (see Fig. 1 in Gunsolus et al. [40]).

### Drawbacks of using SaO_2_ to estimate oxygen delivery

As with SpO_2_, high or low SaO_2_ values do not necessarily signify sufficient or insufficient convective DO_2_ to the brain. In regard to diffusive DO_2_, as already noted, large decreases in PaO_2_ to the right of the upper inflection point of the oxygen-dissociation curve cause only small changes in SaO_2_. This limits the usefulness of SaO_2_ readings in identifying decreases in diffusive DO_2_(Fig. 5). This is compounded by right or left shifts of the oxygen-dissociation curve. The curve shifts to the right (a lower SaO_2_ is required to achieve a given PaO_2_) with acidosis, increases in PCO_2_, 2,3-diphosphoglycerate, with certain hemoglobinopathies and fever [44, 45]. With the latter, a common occurrence in many critically ill patients, any given PaO_2_ will be associated with a lower SaO_2_ [45]. At a temperature of 37 °C, a PaO_2_ of 60 mm Hg (at normal pH and PaCO_2_) will be accompanied by an SaO_2_ of 91.1%. Temperature elevation to 40 °C will produce an SaO_2_ of 85.8% (5.3% decrease) [46].

The oxygen-dissociation curve shifts to the left (a higher SaO_2_ is required to achieve a given PaO_2_) with the inverse of the physiological factors listed above, and also with fetal hemoglobin and carbon monoxide intoxication. A leftward shift in the curve, which is inevitable with a decrease in arterial carbon dioxide pressure (PaCO_2_), a common occurrence in hypoxic patients [45] means that small decreases in SaO_2_ (and SpO_2_) are associated with large decreases in PaO_2_ [47].

## Intubation criteria based on PaO_2_ thresholds

Investigators have proposed instituting invasive ventilation when PaO_2_ is less than 65 mm Hg [48], 60 mm Hg [13, 49], 50 mmHg [37, 50], and 45 mm Hg [17].

### Drawbacks of using PaO_2_ to estimate oxygen delivery

PaO_2_ is only an indirect indicator of CaO_2_. Accordingly, it gives limited insight into convective DO_2_. Capillary PO_2_ is the driving pressure for O_2_ to diffuse into the cells (Fig. 3), and results from an interplay of PaO_2_, convective DO_2_, oxygen consumption and shifts in the oxygen dissociation curve [44, 51–53]. In other words, even a normal (or near normal) PaO_2_ does not automatically guarantee sufficient diffusive DO_2_ when tissue perfusion is reduced (stagnant hypoxia), or with anemia (anemic hypoxia) and when the mitochondria are unable to make use of oxygen (histotoxic hypoxia) [52–58].

## Intubation criteria based on PaO_2_/FiO_2_ thresholds

In hypoxemic patients investigators recommend intubation when the arterial-to-inspired oxygen (PaO_2_/FiO_2_) ratio is less than 200 [12, 59], 100 [18], or 85 [60].

### Drawbacks of using the PaO_2_/FiO_2_ ratio

Accurate recordings of PaO_2_ are easily obtainable. In contrast, the variable entrainment of ambient air during oxygen supplementation in most non-intubated patients makes it impossible to know with certainty the FiO_2_ reaching the trachea [61, 62]. For instance, a high-flow oxygen system through nasal cannula set at a flow of 50 L·min^− 1^ and an FiO_2_ of 60% generates an FiO_2_ anywhere between 35% and 60% [62] – the result is an underestimation of the true PaO_2_/FiO_2_ ratio. Such underestimation may induce intensivists to intubate patients who are not hypoxemic. The confounding factor of ambient air entrainment is underscored by the observation that placement of a surgical mask in patients receiving high-flow oxygen through nasal cannula increased PaO_2_ an average of 20 mm Hg [63].

Another drawback of the PaO_2_/FiO_2_ ratio stems from the curvilinear relationship between PaO_2_ and FiO_2_ that varies with the degree of ventilation–perfusion inequality and shunt [64, 65]. For instance, in patients with ARDS and a fixed shunt, alterations in FiO_2_ caused PaO_2_/FiO_2_ to fluctuate unpredictably by greater than 100 mmHg [66]. In patients who fulfil all ARDS criteria, administration of 100% oxygen for 30 min caused PaO_2_/FiO_2_ to increase such that 58.5% were no longer categorized as ARDS [67].

Regarding convective and diffusive DO_2_, PaO_2_/FiO_2_ ratio plays no role in any biological process and is misleading in the assessment of oxygen physiology [64, 65]. For example, Yarnell and Brochard *(who agree we quote their personal communication, August 12, 2023)*, reported that the unadjusted hospital mortality on day-28 of over two thousand seven hundred patients with acute hypoxemic respiratory failure of non-COVID-19 patients who were never intubated was not greater than the mortality of patients intubated within 3 hours or after 3 hours after meeting a PaO_2_/FiO_2_ ratio threshold of less than 80, 100 or 150. The investigators advise caution as results can vary across centers and patient groups. The same investigators also computed the saturation-to-inspired oxygen (SF) ratio in the same cohort of patients [68]. Then, they performed an adjusted analysis and concluded that different SF ratio thresholds for intubation “can either increase or decrease the expected mortality, with the direction of effect likely depending on baseline mortality risk and clinical context”.

## Intubation criteria based on CaO_2_ thresholds

In 2021, Voshaar et al. [69] proposed a therapeutic strategy that calls for invasive ventilation when hypoxemic patients with severe COVID-19 pneumonia and presumed normal cardiac function had a CaO_2_ of less than 9 mL O_2_ ∙ 100^-1^ mL of blood despite implementation of noninvasive oxygenation strategies. In that non-randomized, retrospective, study conducted in 78 patients admitted in two German hospitals, the mean (± SD) nadir in SpO_2_ was 84.4 ± 6.5%. Overall mortality was 7.7%, which was three times lower than the mortality of patients hospitalized with severe COVID-19 pneumonia in Germany [70].

### Drawbacks of using CaO_2_ as an intubation criteria

The proposed CaO_2_ threshold of 9 mL O_2_ ∙ 100^− 1^ mL of blood is based on calculations made from two isolated observations, one in healthy subjects [71] and the other in anesthetized, paralyzed healthy piglets [72]. Yet, for this threshold to be an appropriate justification to escalate therapy, several major assumptions must be made. First, CaO_2_ must be a valid estimate of diffusive and convective DO_2_. Next, one must assume that the value of global convective DO_2_ below which oxygen consumption becomes delivery-dependent (critical DO_2_) is known and that global critical DO_2_ and brain’s regional critical DO_2_ have identical values. Finally, the physiologic effects of a decrease in CaO_2_ are independent from the mechanism that caused that decrease. Unfortunately, these assumptions are either incorrect or have not been tested.

### Drawbacks of using CaO_2_to estimate oxygen delivery

CaO_2_ gives incomplete information about diffusive and convective DO_2_. Accordingly, unless critically decreased, CaO_2_ cannot inform the physician about a patient’s cerebral oxygen supply.

A given CaO_2_ results from a combination of a myriad of Hb and SaO_2_ values. For example, a Hb concentration of less than 7 g/dL is a common threshold for transfusion of red blood cells [73, 74]. When Hb is 7 g/dL and the diffusion pressure – or PaO_2_ – is in the normal range of 80 to 95 mm Hg and the corresponding SaO_2_ is 95 to 97%, the CaO_2_ will range from 9.4 to 9.6 mL O_2_ ∙ 100^-1^ mL of blood (Fig. 5). This is a situation which most patients can safely tolerate [73, 74]. To achieve similar CaO_2_ values when Hb is 15 g/dL, PaO_2_ has to decrease to 25.2 to 25.5 mm Hg and SaO_2_ has to decrease to 45.8 to 46.5% (Fig. 5). With only few exceptions (see below), these values of PaO_2_ and SaO_2_, even in healthy subjects, cause loss of consciousness, myotonic twitches, and convulsions [6]. In other words, CaO_2_ gives no direct information about oxygen supply to the brain limiting its utility in informing a decision to intubate the individual patient.

**Fig. 6:**
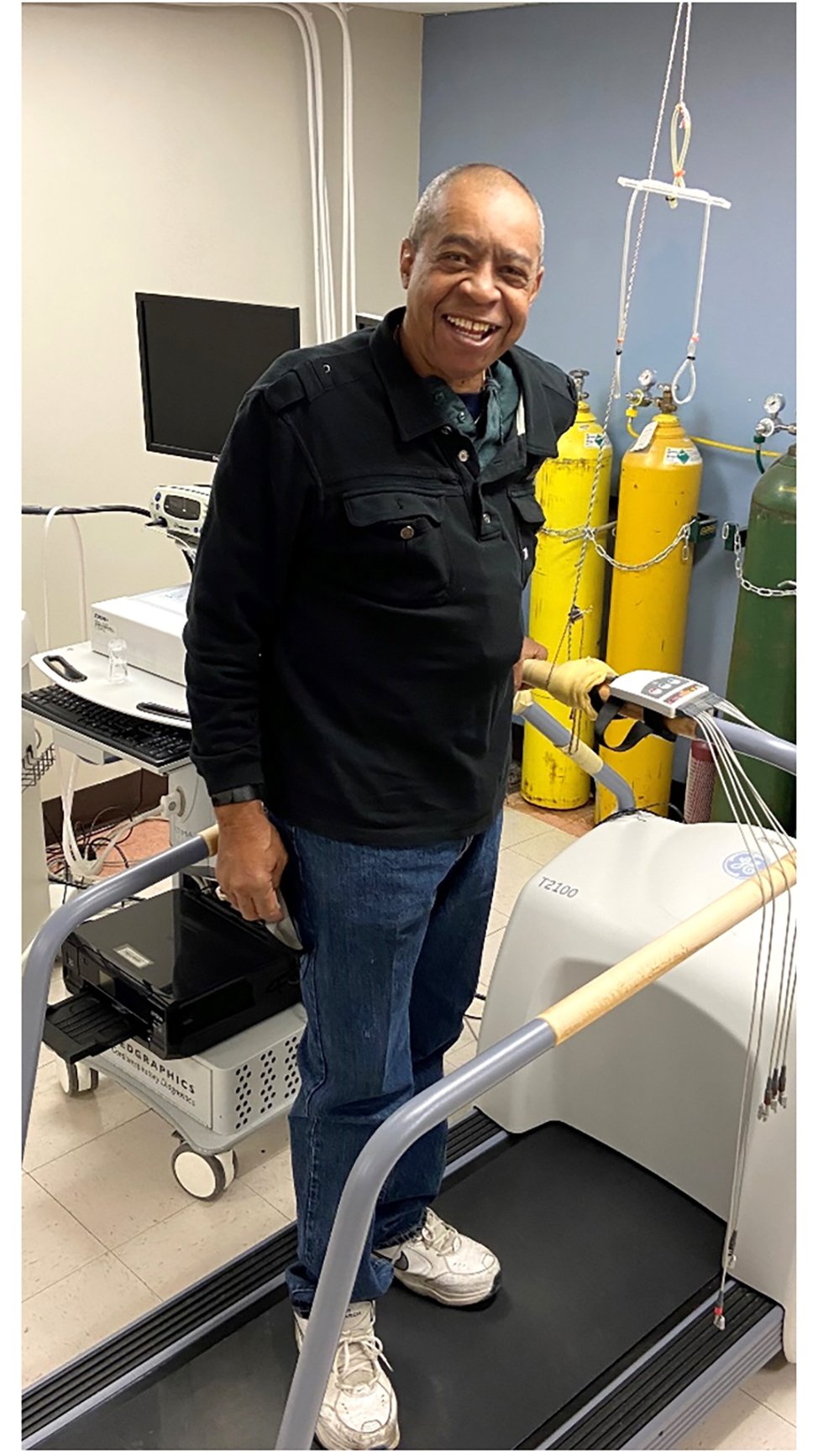
Index patient after completing twelve weeks of pulmonary rehabilitation after hospital discharge

## Physiologic approach to intubation

Basing the decision to intubate hypoxemic patients on physiologic principles requires knowledge of the minimal oxygen supply to maintain a tissue PO_2_ capable to sustain the oxidative metabolism of the brain. Although uncertain, such critical tissue PO_2_ is probably about 20 mmHg or less [75, 76]. In a healthy subject at rest, the mean oxygen consumption of the brain is about 46 mL.min^− 1^ and its blood flow is about 620 mL.min^− 1^ [7]. This corresponds to an arterial-to-venous oxygen content difference of 7.4 mL O_2_ ∙ 100^− 1^ mL of blood when hemoglobin and pH are within normal values [7]. The lowest CaO_2_ to secure such difference in oxygen content while maintaining a venous PO_2_ (and by implication a cerebral PO_2_ [7]) greater than 20 mm Hg is 13.8 mL O_2_ ∙ 100^− 1^ mL of blood. This corresponds to a PaO_2_ of 36 mm Hg and SaO_2_ of 68% [7]. This is the oxygenation experienced by tourists on drives to the top of Mount Evans (4350 m) for prolonged periods; many are comfortable, whereas some sense dyspnea [77]. It is important to note that the above computations are oversimplifications as they ignore hypoxia-induced increases in Qc and hyperventilation-induced hypocapnia with its associated cerebral vasoconstriction [78]. They also ignore the complex mechanisms that regulate cerebrovascular reactivity, cerebral metabolism during hypoxia [56, 78] and the large inter-individual variation in tolerance to hypoxia [6]. Accordingly, there is no simple answer to the question: what is the safe lower limit of PaO_2_ or SaO_2_ or CaO_2_? For example, a PaO_2_ of 36 mm Hg (and accompanying SaO_2_ of 68%), will be insufficient to sustain the oxidative metabolism of the brain in a patient who is anemic [54]. It will also be insufficient when cerebral perfusion is sub-optimal such as in patients with pre-existent cerebrovascular disease [79], decreased cardiac output [79], insufficient mean arterial blood pressure [54, 80] or cerebral vasoconstriction induced, for instance, by acute hypocapnia [6, 54]. At the same time, a PaO_2_ as low as 25 mmHg, with a corresponding SaO_2_ of about 45%, can ensure consciousness and by implication the oxidative metabolism of the brain in acclimatized mountaineers [81], and in patients with acute-on-chronic respiratory failure [82]. These are situations associated with compensatory polycythemia [81], maximal cerebral vasodilatation [7] and cellular adaptations to hypoxia [83].

How can we inform our decision to intubate a hypoxemic patient who is not in apparent respiratory distress? Considering the uncertainties about the critical cerebral PO_2_ [7, 76, 84], the non-uniform cerebral distribution of PO_2_ and oxygen demands [80, 85], the complex mechanisms that regulate cerebrovascular reactivity and cerebral metabolism during hypoxia [56, 78], the technical difficulties in monitoring cerebral PO_2_ [84] and the dangers associated with a liberal approach to insert an endotracheal tube [2] and institute invasive ventilation [3, 4], we see the decision of when to insert an endotracheal tube as one of the most challenging faced by any intensivist. Cognizant of these uncertainties, we consider intubation when our hypoxemic patient in neither apparent respiratory distress (operationally defined as the clinically observable corollary of dyspnea based on a patient’s display of physical/clinical signs) nor in shock becomes cognitively impaired suggesting emergent cerebral hypoxia [6]. When deciding to intubate, we also consider additional factors including blood pressure, and estimations of cardiac function, peripheral perfusion, CaO_2_ and its determinants. These additional factors, with all their limitations, are the indices we use in hypoxemic patients with coexistent pathologies that themselves cause cognitive impairment. In such cases, it remains to be determined whether computing the Intensive Care Unit Respiratory Distress Observation Scale or IC-RDOS, developed to assess dyspnea in critically ill patients who cannot easily communicate [86], may be helpful. We also recognize that whether the decision to institute invasive ventilation should be more liberal in patients with greater predicted mortality and less liberal in patients with lower predicted mortality is unknown [68].

## Intubation of hypoxemic patients: Nosology, tacit knowledge, and the future

The focus of this physiologic review is the intubation of the hypoxemic patient is neither in apparent respiratory distress nor shock. There is a paucity of research that evaluates this group of patients – and no randomized controlled trials. Unfortunately, the nosology of the disease entity under consideration and the crucial contribution of tacit knowledge in medical decision making, make the design and the applicability of such hypothetical randomized controlled trials dubious if not impossible.

### Nosology and clinical trials

Nosology is the branch of medical science dealing with the classification of diseases. Some diseases can be classified in etiologic or causal terms (e.g., Legionnaires’ disease) [5]. Diseases classified in etiologic or causal terms allow for complete scientific rigor [5]. This contrasts with syndromes (e.g., ARDS) or clinical entities (e.g., a patient in respiratory distress who is ‘tiring out’), which are defined by way of a description of symptoms and signs [5]. Hypoxemia in a patient in no apparent respiratory distress reflects a disease entity with indistinct boundaries. This has crucial implications in the design of clinical trials of these patients. When designing such a hypothetical trial, investigators must come up with a list of inclusion and exclusion criteria that serve as nodal points, which must be diligently (rigidly) followed to ensure the internal consistency of the study [87]. As discussed in this review, however, the soundness of oxygenation thresholds is fundamentally imprecise. Even if such a hypothetical study were undertaken, how would the clinician implement its results in his or her clinical practice?

### Tacit knowledge and clinical decision making

In the context of our hypothetical study, hypoxemic respiratory failure can be defined without making any subjective value judgement – i.e., investigators would be required to rigidly follow a priori inclusion and exclusion criteria. This strategy, however, does not reflect bedside clinical decision making [87]. When making decisions about the treatment of an individual patient it is not possible to avoid subjective value judgments (things being assessed on a scale of goodness or badness) [5, 88]. Physicians base the decision to intubate on their clinical gestalt. Physicians may not be able to articulate the precise reasons behind this decision in the form of words [89]. This is because a wise physician standing at a patient’s bedside senses a great deal of worthwhile information—much more than can be expressed in words [5]. In short, there is a very large tacit coefficient to clinical knowledge—physicians know much more than they can communicate verbally [89, 90]. There is an enormous difference between the assessment made by an experienced physician standing at a bedside and the assessment the same physician makes on hearing information (about the same patient) relayed over the telephone by a junior resident [5]. An experienced and wise physician employs intuition rather than explicit rules in deciding what is best for a particular patient in a particular setting [5]. The practice of clinical medicine at the bedside involves cognitive processes and skill performances that cannot be incorporated into randomized controlled trials or observational research studies. A physician who regards such intuition as unscientific and thus flawed demonstrates a fundamental misunderstanding of both the epistemology of science and of the nature of clinical practice [89].

## Conclusion

It is not possible to articulate the indications for mechanical ventilation in the individual patient in the form of a list of items. In clinical practice, the decision to insert an endotracheal tube is based, rather, on clinical judgement, gestalt, and tacit knowledge [5]. Our failure to formulate a list of indications does not mean that we advocate a laissez-faire approach to instituting mechanical ventilation. For instance, earlier we mentioned the limitations of PaO_2_ in informing us on the patients’ cerebral oxygen supply. This does not mean that we consider PaO_2_ unimportant. When we learn that a patient is acutely and persistently hypoxemic despite implementation of noninvasive oxygenation strategies, we immediately consider steps to institute invasive ventilation. But it is not possible to pick a PaO_2_ breakpoint at which the benefits of invasive ventilation will decidedly outweigh its hazards across all patients. It is futile to imagine that decision making about instituting invasive ventilation can be condensed into an algorithm with numbers at each nodal point. In sum, an algorithm cannot replace the presence of a physician well skilled in the art of clinical evaluation who has a deep understanding of pathophysiologic principles [5].

## Coda

*Our index patient spent 38 days in the hospital, 16 of which in the intensive care unit. Despite SpO*_*2*_*in the 80s% (and occasionally in the 70s%) while on high-flow oxygen through nasal cannula, he never developed respiratory distress. His mentation remained normal; he attentively watched television and appropriately conversed with family using his cell phone. He was not intubated. The patient was discharged home on 2 L·min*^*− 1*^*oxygen. Supplemental oxygen was discontinued one month after discharge. He successfully completed outpatient pulmonary rehabilitation*(Fig. 6).

## Data Availability

This is a Review paper. The sources for the review are listed in the reference list and are available on PubMed.

## Abbreviations

∆PO_2_: Gradient in oxygen pressure
CaO_2_: Arterial blood oxygen content
DO_2_: Oxygen delivery
Hb: hemoglobin
HbO_2_: Oxyhemoglobin
PaCO_2_: Arterial carbon dioxide pressure
PaO_2_/FiO_2_: Arterial-to-inspired oxygen ratio
PaO_2_: Arterial oxygen pressure
PO_2_: Partial pressure of oxygen
Qc: Cardiac output
SaO_2_: Arterial oxygen saturation
SD: Standard deviation
SF: Saturation-to-inspired oxygen ratio
SpO_2_: Peripheral oxygen saturation
